# Emerging (val)ganciclovir resistance during treatment of congenital CMV infection: a case report and review of the literature

**DOI:** 10.1186/s12887-017-0933-6

**Published:** 2017-08-22

**Authors:** Beatriz Morillo-Gutierrez, Sheila Waugh, Ailsa Pickering, Terence Flood, Marieke Emonts

**Affiliations:** 1 Paediatric Infectious Diseases and Immunology Department, Great North Children’s Hospital, Queen Victoria Road, Newcastle upon Tyne, NE1 4LP UK; 2 Microbiology Department, Freeman Hospital, Newcastle upon Tyne, NE7 7DN UK; 3 Institute of Cellular Medicine, Newcastle University, Newcastle upon Tyne, UK

**Keywords:** Cytomegalovirus, Congenital, Resistance, Valganciclovir, Ganciclovir, Case report

## Abstract

**Background:**

Congenital cytomegalovirus (cCMV) infection is an important illness that is a common cause of hearing loss in newborn infants and a major cause of disability in children. For that reason, treatment of symptomatic patients with either ganciclovir or its pro-drug valganciclovir is recommended. Treatment duration of 6 months has been shown to be more beneficial than shorter courses; however, there is uncertainty regarding emergence of resistance strains, secondary effects and long term sequelae.

**Case presentation:**

Here we present a female infant with symptomatic cCMV who was treated from day 5 of life with oral valganciclovir. In spite of close monitoring of her drug levels and increments of her treatment dose according to weight gain, she developed ganciclovir resistance after 4 months of treatment, with increasing viraemia and petechiae. Adherence to treatment was assessed and felt to be good. Clinically, although she had marked developmental delay, she was making steady progress. In view of the development of resistance treatment was stopped at 5 months of age. No secondary effects of ganciclovir were noted during the whole course.

**Conclusions:**

There were few cases in the literature reporting resistance to ganciclovir for cCMV before the new recommendations for a 6 months treatment course for this infection were published. As demonstrated in our patient, surveillance with periodic viral loads and drug monitoring are vital to identify emerging resistance and optimise antiviral dosing according to weight gain.

## Background

Congenital cytomegalovirus (cCMV) infection is an important illness that is a common cause of hearing loss in newborn infants and a major cause of disability in children. A recent evidence based guideline [1] recommends the use of ganciclovir, which is given intravenously, or its pro-drug, oral valganciclovir, in symptomatic patients. Currently a 6 month rather than 6 week course of valganciclovir is advocated as it has shown more clinical benefits with no increase in secondary effects [2, 3]. However, as frequent monitoring of drug levels and CMV viral load is not routine practice for cCMV management in immunocompetent patients in most centres, there is a lack of information regarding emergence and sequelae of resistance mutations. To date, not many cases have been described in the literature; the first case was born prematurely with hydrops foetalis. The infant died at 113 days of life, having failed to respond to treatment. Although the information is limited, resistance mutations seemed to be present very early on, but the proportion of resistant strain increased over time as detected by pyrosequencing [4]. The second case, reported by Campanini et al. [5], was of a symptomatic newborn diagnosed at birth with cCMV who developed multidrug resistance, including to ganciclovir, with an increase in symptoms. The third case was a preterm infant with symptomatic cCMV infection who presented with 5 mutations associated with ganciclovir resistance after 120 days of treatment with (val)ganciclovir [6]. Unfortunately no virological information was available before that point. The fourth case was described by Choi et al. [7], in a patient who developed (val)ganciclovir resistance noted as an increment in viraemia with no associated symptoms; the viraemia cleared after stopping the treatment. This patient developed neutropenia while on ganciclovir.

## Case presentation

We report a 6 month-old female infant, first daughter of a healthy, young couple. The pregnancy was followed up for microcephaly detected by prenatal ultrasound. The patient was born by caesarean section for pathological cardiotocography following premature rupture of membranes at 35 weeks; the birth weight was 2.06 kg (25th centile), and the head circumference was 30 cm (9th centile).

At birth, in addition to microcephaly, she was noted to have petechial rash, hepatosplenomegaly and respiratory distress requiring CPAP for the first 48 h of life. The platelet count was 44 × 10^3^/μL and ALT was 6 IU/L. CMV was detected by PCR in blood and urine on day 3 of life with a CMV DNA level in blood of 3.6 × 10^5^ copies/mL. Ophthalmology assessment on day 4 of life showed normal retinae bilaterally, and audiology screening on day 5 showed left sensorineural hearing loss (60 dB). The MRI showed periventricular and multiple thalamostriate areas of calcification.

In view of the symptomatic manifestations of cCMV infection, the patient was started on valganciclovir (16 mg/kg twice daily, commercially available solution) on day 5 of life.

Of note she was on home oxygen during the first months and developed apnoeic episodes from day 27 of life and on one occasion required intubation and mechanical ventilation for less than 24 h following a respiratory arrest. There was absence of gag reflex, as a probable manifestation of congenital CMV, and the child was fed via nasogastric tube.

**Fig. 1:**
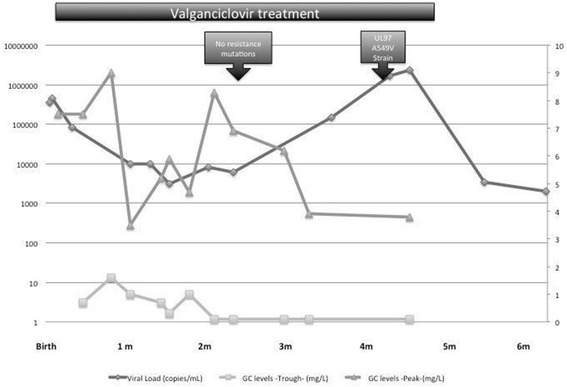
Timeline showing viral loads and ganciclovir (GC) levels measurements, as well as the determination of mutations

Her viral loads were measured periodically in blood as well as her trough and peak ganciclovir levels (Fig. 1). Initially there was a reduction in CMV viral load. Her valganciclovir dose was increased according to her weight gain and drug levels and there were no concerns regarding compliance.

Clinically, she was making steady progress, specifically in her weight gain and neurodevelopment, although there were some inter-current episodes of respiratory deterioration coincidental with flaring up of the petechial rash with normal platelet counts.

After 4 months of treatment, an increase in viral load was noted reaching a peak of 3.6 × 10^6^ copies/mL, as seen in the Fig. 1. Her valganciclovir had been maximised up to 140% the standard treatment dose because of low drug levels and no signs of toxicity.

Blood was therefore sampled for viral nucleotide sequencing analysis (Manchester Medical Microbiology Partnership), which confirmed the presence of the A594V mutation in the CMV UL97 gene conferring resistance to ganciclovir. No mutations were detected in the UL54 gene, inferring sensitivity to foscarnet and cidofovir. The retrospective analysis of a sample when the patient was 2 months of age identified no known CMV resistance mutations.

Her full blood count, liver function tests and electrolytes remained within normal limits.

Valganciclovir was therefore stopped at 5 months of age. In view of her steady improvement, no benefit was felt in starting any of the other alternative drugs, such as foscarnet or cidofovir, which are associated with significant toxicity, have limited CNS penetration and require intravenous administration. There were no signs of primary immunodeficiency, including a normal lymphocyte subset panel, and her CMV level taken when stopping treatment had decreased to 3.5 × 10^3^ copies/mL. In addition, her IgM and IgG, both negative at 4 months, were found to be positive, with high avidity IgG. One month after the end of treatment her viral load continued to decrease, with a level below 2 × 10^3^ at 6 months of age, and was undetectable at 9 months of age. At 2.5 years of life CMV was not unexpectedly still detected in urine, but levels were too low for reliable mutation analysis. In spite of her marked developmental delay she was still making some steady progress. She can pull herself to stand, and sit unaided, but has no saving reflex when she falls, and she can grasp and transfer toys. Vision is normal, she does not require hearing aids, and she is vocalising, but uses no single words. She developed severe epilepsy at about 2 years of life..

## Conclusions

To date, this is the fifth case of cCMV resistant to ganciclovir described in the literature while on treatment. The pro-drug valganciclovir was used throughout treatment. Her weight and drug levels were closely monitored, with subsequent increments of the valganciclovir dose, aiming for 0.5–1.0 mg/L and 7–9 mg/L for trough and peak levels respectively. Of note, before emergence of the resistant strain, the patient had a period of suboptimal levels in spite of having her dose maximised. Although poor adherence cannot be fully excluded, this observation may reflect individual variation in the pharmacokinetics of valganciclovir [8]. There were no signs of malabsorption. Fortunately, she did not present any adverse effect such as neutropenia, thrombocytopenia or renal toxicity.

At the time CMV resistance was identified clinical progress was steady despite the rise in viral load. Once the resistant strain was detected, a decision was made to stop her treatment. In subsequent follow up, she seroconverted to IgG with high avidity and her viral load declined, showing an effective immune response to control the infection. We postulate that the rise in the viral load due to the resistance resulted in an increased immune stimulus to CMV, acting as a boost to immunity and, subsequently, a fall in the viral load.

This case adds another example of cCMV and highlights the importance of frequent monitoring to detect resistant strains, as well as adverse effects related to treatment. As treatment for symptomatic cCMV has been recommended with (val)ganciclovir (1), the emergence of resistance mutations is a potential risk given the combination of high viral loads, prolonged treatment, and the potential for suboptimal drug levels even with dose increments in newborns and infants [8–10]. This risk of resistance increases with longer durations of treatment, which is of relevance given recent evidence advocating a 6 month treatment course in light of slightly improved outcome in terms of language and receptive communication [2, 3]. A risk of 5–10% for emergence of resistance has been suggested with long term ganciclovir therapy in transplant recipients [9]. Because recommendations for 6 months treatment for cCMV are new, surveillance to identify emerging resistance and optimisation of antiviral dosing accounting for weight-changes and therapeutic drug monitoring are vital^1^. Once weekly or fortnightly trough and peak levels (2 h post dose) until stabilisation would be recommended. Intervals could then be increased provided the dose is increased weekly with weight gain. This could however, be monitored remotely from home, or via the GP or community service if needed. Finally, it is important to remember that the risk of infection for cCMV can be decreased following simple hygiene measures such as hand washing after nappy changes or wiping a child’s nose [11–13]. Health care professionals should ensure that pregnant woman are aware of the importance of these measures.

## Abbreviations

cCMV: Congenital cytomegalovirus

## Footnotes

1 After the introduction of the recommendations of (val)ganciclovir treatment for symptomatic congenital CMV, surveillance with periodic viral loads and drug monitoring are vital to identify emerging resistance and optimise antiviral dosing in line with weight gain.
